# DNA methylation changes related to nutritional deprivation: a genome-wide analysis of population and in vitro data

**DOI:** 10.1186/s13148-019-0680-7

**Published:** 2019-05-16

**Authors:** Yujie He, Lot D. de Witte, Lotte C. Houtepen, Danny M. Nispeling, Zhida Xu, Qiong Yu, Yaqin Yu, Elly M. Hol, René S. Kahn, Marco P. Boks

**Affiliations:** 1 Brain Center University Medical Center Utrecht, Department of Psychiatry, Utrecht University, A01.468, PO Box 85500, 3508 GA Utrecht, The Netherlands; 2 Department of Psychiatry, Icahn School of Medicine at Mount Sinai, New York City, USA; 3 Department of Epidemiology and Biostatistics, School of Public Health, Jilin University, Changchun, China; 4 Brain Center University Medical Center Utrecht, Department of Translational Neuroscience, Utrecht University, Utrecht, The Netherlands; 5 Department of Neuroimmunology, Netherlands Institute for Neuroscience, an Institute of the Royal Academy of Arts and Sciences, 1105 BA Amsterdam, The Netherlands

**Keywords:** Chinese famine, Nutrition deprivation, Genome-wide DNA methylation, Pathway analysis

## Abstract

**Background:**

DNA methylation has recently been identified as a mediator between in utero famine exposure and a range of metabolic and psychiatric traits. However, genome-wide analyses are scarce and cross-sectional analyses are hampered by many potential confounding factors. Moreover, causal relations are hard to identify due to the lack of controlled experimental designs. In the current study, we therefore combined a comprehensive assessment of genome-wide DNA methylation differences in people exposed to the great Chinese famine in utero with an in vitro study in which we deprived fibroblasts of nutrition.

**Methods:**

We compared whole blood DNA methylation differences between 25 individuals in utero exposed to famine and 54 healthy control individuals using the HumanMethylation450 platform. In vitro, we analyzed DNA methylation changes in 10 fibroblast cultures that were nutritionally deprived for 72 h by withholding fetal bovine serum.

**Results:**

We identified three differentially methylated regions (DMRs) in four genes (*ENO2*, *ZNF226*, *CCDC51*, and *TMA7*) that were related to famine exposure in both analyses. Pathway analysis with data from both Chinese famine samples and fibroblasts highlighted the nervous system and neurogenesis pathways as the most affected by nutritional deprivation.

**Conclusions:**

The combination of cross-sectional and experimental data provides indications that biological adaptation to famine leads to DNA methylation changes in genes involved in the central nervous system.

**Electronic supplementary material:**

The online version of this article (10.1186/s13148-019-0680-7) contains supplementary material, which is available to authorized users.

## Background

DNA methylation is one of the epigenetic mechanisms that plays an important role in the cellular responses to detrimental environmental influences that are involved in the etiology of many diseases [1]. Studies show that early life exposure to nutritional deprivation is associated with stable DNA methylation differences [2, 3]. Nutritional deprivation, especially in utero and early in life, has detrimental effects on human development and significantly increases the risk of multiple chronic diseases later in life [3–6].

A seminal example of the impact of in utero exposure to nutritional deprivation is the cohort study on offspring from mothers that were pregnant during the Dutch hunger winter during the Second World War, which was intense and well-documented but with brief duration [7]. This study identified persistent differential methylation of the insulin-like growth factor II (IGF2), as a key human growth and development factor involved in the response to famine in utero [3]. Subsequent studies of this cohort identified DNA methylation changes as mediators of the association between maternal famine and metabolic disease in adulthood [6, 8]. Other epigenetic differences associated with famine exposure in utero have been related to schizophrenia [9] and type 2 diabetes [10].

While the Dutch famine is the most extensively studied famine in the literature, the Chinese great famine (1959–1961) was one of the largest famines recorded around the world and had more severe consequences resulting in an estimated 30 million deaths [11]. The offsprings of those mothers who suffered famine were shorter in length [5], had worse midlife health [12], and had a higher rate of chronic diseases [13, 14]. Studies also showed a twofold increased risk to develop schizophrenia among offspring conceived at the height of the famine [15, 16]. However, only one genome-wide DNA methylation study is reported in the Chinese famine population [17]. To further understand the impact of maternal famine on DNA methylation changes in offspring, we compared genome-wide DNA methylation from whole blood of Chinese participants exposed to famine in the first trimester to unexposed controls from the same populations.

Since a cross-sectional population-based study is subject to residual confounding and does not allow the examination of the direct effect of nutritional deprivation, we subsequently performed an in vitro study of human fibroblasts before and after exposure to nutritional deprivation. By combining the result of a genome-wide methylation approach of both studies, we aim to provide an unbiased investigation of DNA methylation changes induced by nutritional deprivation.

## Methods

### Chinese famine sample

The sample of Chinese famine is part of our previous study and has been described in more detail elsewhere [9]. In short, volunteers were recruited in the northern province of Jilin, China. Considering the almost complete penetration of famine during January 1960 and September 1961, it is assumed that those born during that period will have been exposed. A total of 79 healthy participants were included of which 25 were exposed to famine during the first 3 months in utero. All participants provided written informed consent. Table 1 gives the full details of the participants.

**Table 1:** Summary of characteristics of the Chinese famine samples

	Unexposed	Exposed to maternal famine
*N*	54	25
Age (sd)	46.8 (1.0)	50.3 (0.5)
Male *N* (%)	21 (39%)	10 (40%)

### Fibroblast in vitro study

The in vitro fibroblast experiment was described in more detail previously [9]. In short, fibroblasts were obtained by skin biopsies from five healthy participants of Dutch descent, of which one was male and four were female (mean age = 38.4 years, sd = 7.0) (see Table 1). All participants provided written informed consent. Fibroblasts were plated in two T25 flasks in Minimum Essential Medium (MEM) (Gibco®) with 15% fetal bovine serum (FBS)(Gibco®) and 1% penicillin-streptomycin PenStrep (Gibco®) and in an atmosphere of 95% atmospheric air and 5% CO_2_ at 37 °C (normal conditions). After reaching 70–80% confluence, the supernatant was removed and the cells were washed three times with phosphate buffered saline (PBS) (BioWhittaker® Reagents, Lonza). Next, one of the T25 flasks from each donor was cultured in the non-famine condition with Minimum Essential Medium (MEM) (Gibco®) supported with 15% FBS, while the other T25 flasks were cultured in only Minimum Essential Medium (MEM) as famine condition. After 72 h, cells were harvested from each flask and stored as cell pellet for DNA isolation.

### DNA processing

DNA from the Chinese famine samples was extracted from whole blood using the Gentra Puregene Kit (Qiagen, Valencia, CA, USA). Fibroblast cell pellets were used for DNA isolation according to the manufacturer’s instructions (Qiagen, Hilden, Germany). The DNA concentration and quality were examined using NanoDrop (Thermo Fisher Scientific, Massachusetts, USA). Bisulfite conversion of each DNA sample was conducted according to the manufacturer’s instructions of the Zymo EZ DNA MethylationTM Kit (Zymo, Irvine, CA, USA). Quality and quantity of the bisulphite treated single stranded DNA was examined using NanoDrop.

### Genome-wide analysis of DNA methylation

One hundred and fifty nanograms of bisulfite-converted DNA from the Chinese famine study was used to quantify genome-wide patterns of DNA methylation using the Illumina Infinium HumanMethylation450 BeadChip. Genome-wide DNA methylation levels of fibroblasts were obtained using Illumina HumanMethylation EPIC BeadChip arrays. For the Chinese famine samples, intensity readouts, beta and *M* value calculation, and cell-type proportion estimates were obtained using the minfi package (version 1.10.2) in Bioconductor [18]. Probes were excluded based on a bead count less than three (*n* = 279 probes) or a detection *p* value larger than 0.001 in at least 5% of the samples (*n* = 2125 probes). Non-autosomal or cross-hybridizing probes were discarded as were loci with SNPs of minor allele frequency larger than 1% within 1 bp of the primer [19]. None of the blood samples had over 1% of failed probes. All 79 DNA samples survived quality control [20], and 397,985 loci were left in the dataset for further analysis. The normalization was performed using the functional normalization procedure which is implemented in the minfi package. Additional adjustments were made using the genetic principal components estimated according to Barfield et al. [19]. Moreover, the blood-based analysis included an adjustment for cell-type (B cells, CD8 T cells, CD4 T cells, natural killer cells, monocytes, and granulocytes) [21].

The quality control for fibroblasts was performed in a similar workflow as the Chinese famine samples but adjusted to the newer EPIC methylation beadchip. The dataset was pre-processed in R version 3.3.1 with the meffil package [22] using functional normalization [23] to reduce the non-biological differences between probes. To account for technical batch variables, pre-processing was performed in a larger dataset (*n* = 80), including DNA samples of other studies that included brain and blood DNA. However, normalization was conducted for the fibroblast samples only. No mismatches between methylation-predicted sex and actual gender were present nor were there samples with outliers on mean of methylated and unmethylated channels. Probes were removed if they failed quality control (a detection *p* value > 0.01 for > 10% of samples (*n* = 4610) or a bead count < 3 for > 10% of samples (*n* = 68)), were non-specific [20], or were one of the SNP probes included on the array for quality control purposes. All 10 fibroblast DNA samples survived quality control, and 862,160 probes were left in the dataset for further analysis.

For both the Chinese sample and fibroblast samples, the level (percentage) of methylation is expressed as a *β* value, ranging from 0 (unmethylated cytosine) to 1 (completely methylated cytosine), but analyses were performed using *M* values (log2 of *β* values), for better statistical validity [24]. To examine the overlap between the results of the two datasets, DMR and pathway analyses were performed for the 397,985 CpGs that were present on the EPIC as well as the 450 k arrays.

### Pathway analysis

We performed Gene Set Enrichment Analysis (GSEA) for the nominal significant CpGs that overlapped from the Chinese famine and fibroblasts samples. SetRank tool was chosen in the current study for GSEA analysis since it could eliminate many false positive hits [25], especially those biased toward neuronal pathways as these genes are much more abundant and larger in size. Gene Ontology (GO), Kyoto Encyclopedia of Genes and Genomes (KEGG), WikiPathways, and Reactome pathway database are included in the SetRank tool.

### Permutation analysis

The significance level of the identified DMRs was confirmed by permutation analysis whereby *p* values were calculated from all potential DMRs with the same number of CpGs throughout the genome. From the fit of the actual identified DMR in this distribution, an empirical *p* value was derived. The probability of finding the number of overlapping DMRs that we presented from all potential matches was established. All these analyses were based on 10,000 permutations.

### Statistical analyses

Statistical analyses were carried out using R [26]. Analysis of the association of DNA methylation with famine in the Chinese famine samples was performed using linear regression with DNA methylation as dependent and famine, age, gender, and cell-type proportion estimates based on the Houseman algorithm [21] as well as the first two DNA methylation-based ancestry principal components as indicators [19]. In addition, similar as previously, we adjusted for the effects of smoking by deriving a proxy for smoking based on methylation levels of CpGs that were previously associated with smoking [27]. For the fibroblast experiment, methylation changes under the famine condition were assessed using Wilcoxon’s paired rank test. The QQ plots were inspected to assess type I error inflation and power (Additional file 1). DMRcate (version 1.4.2) was used to identify differentially methylated regions (DMRs). Nominal significance for the DMR analysis was set at 0.01 [28]. Only DMRs with the same direction of effect (hyper- or hypomethylation) in both samples were considered overlapping.

## Results

### Identification of differentially methylated regions

Analysis of single CpG methylation did not identify significant differences after adjustment for multiple testing due to insufficient power. The QQ plot indicated the analysis was underpowered to detect genome-wide differentially methylated probes (Additional file 1 shows the QQ plots). Additional file 2 provides the information and test statistics of the nominally associated loci (18,871 for the Chinese famine and 56,375 for the fibroblast experiment). Two thousand seven hundred six CpGs overlapped between nominally associated loci of both experiments. The probability to end up significant in both analyses was higher for CpGs from the famine study (chi-squared = 843.97, df = 1, *p* value < 0.001) as a logical result from the larger number of loci on the methylation array of the fibroblast experiment. However, the odds of identification as nominal significant was also significantly larger in the famine study (chi-squared = 1398.4, df = 1, *p* < 0.001) most likely as a result of a larger power. Analysis of DMRs in the Chinese famine cohort identified 613 different methylated regions (DMRs) and 1080 DMRs in fibroblast samples. Among these significant DMRs, three DMRs were similarly associated (significant and same direction of effect) in both samples. The three replicated DMRs are all hypomethylated in relation to famine exposure and highlight four gene promoters: DMR1, enolase 2 (*ENO2*) (cg08003732, cg13334990, cg18912645, cg19720347), and DMR2, zinc finger protein 226 (*ZNF226*) (cg19331658, cg03559973, cg19836894, cg19599862, cg03573702). DMR3 is related to 2 gene promoters: coiled-coil domain containing 51 (*CCDC51*) and translation machinery associated 7 homolog (TMA7) (cg00329014, cg06625258, cg07744328, cg01538982, cg24981564, cg12370248, cg07095599, cg11196693, cg03629318, cg15853329, cg21856689, cg26094714, cg25858682). The study from Hannon et al. [29] was used as a lookup for the relation between methylation in blood and brain for the identified loci. cg08003732 and cg13334990 loci in *ENO2* gene were all significantly correlated between blood and four brain regions: the prefrontal cortex (PFC), entorhinal cortex (EC), superior temporal gyrus (STG), and cerebellum (CER). Other loci with significant correlation between blood and brain were cg19331658 in *ZNF226* and cg26094714 in *CCD51*/*TM7* that were correlated with PFC, cg18912645 in *ENO2* and cg12370248 and cg15853329 in *CCD51*/*TM7* that were correlated with EC, and blood cg19720347 methylation in *ENO2* that was correlated with CER. Table 2 shows the characteristics of the DMRs consistently associated to famine in both experiments. Permutation analysis confirmed the significance of most of the presented associations with the exemption of the association of ENO2 to famine in the Chinese sample that showed an empirical *p* value of *p* = 0.099, although combined *p* values of both analyses remain significant (*p* = 0.0016). Additional file 3 presents the results of the permutation analysis.

**Table 2:** Three DMRs consistently associated with famine in both experiments (Chinese famine samples and fibroblasts samples)

DMRs	Gene promoters	CHR	Region (hg19)	CpG numbers	*β* value (Chinese)	*p* value (Chinese)	*β* value (Fibroblasts)	*p* value (Fibroblasts)
DMR1	*ENO2*	chr12	7023752–7024121	4	− 0.0243	1.19E−04	− 0.1523	7.42E−04
DMR2	*ZNF226*	chr19	44669146–44669354	5	− 0.0636	9.21E−03	− 0.3155	1.07E−03
DMR3	*CCDC51*	chr3	48481268–48481793	13	− 0.0290	7.87E−04	− 0.2318	1.25E−06
	*TMA7*							

*DMR* differentially methylated regions, *CHR* chromosome, *hg19* human genome version 19. The first column of the table shows the DMR identifier and followed by the gene name which belongs to the DMR. The chromosome of the gene is provided and followed by the more precise region in hg19 (human genome version 19). The number of significant CpGs response to nutrition deprivation in both studies is presented, and *β* value and *p* value of DMRs in both studies are also presented. *β* value in each study refers to the mean *β* values of identified CpG in each DMR

### Pathway analysis of identified CpG loci

**Fig. 1:**
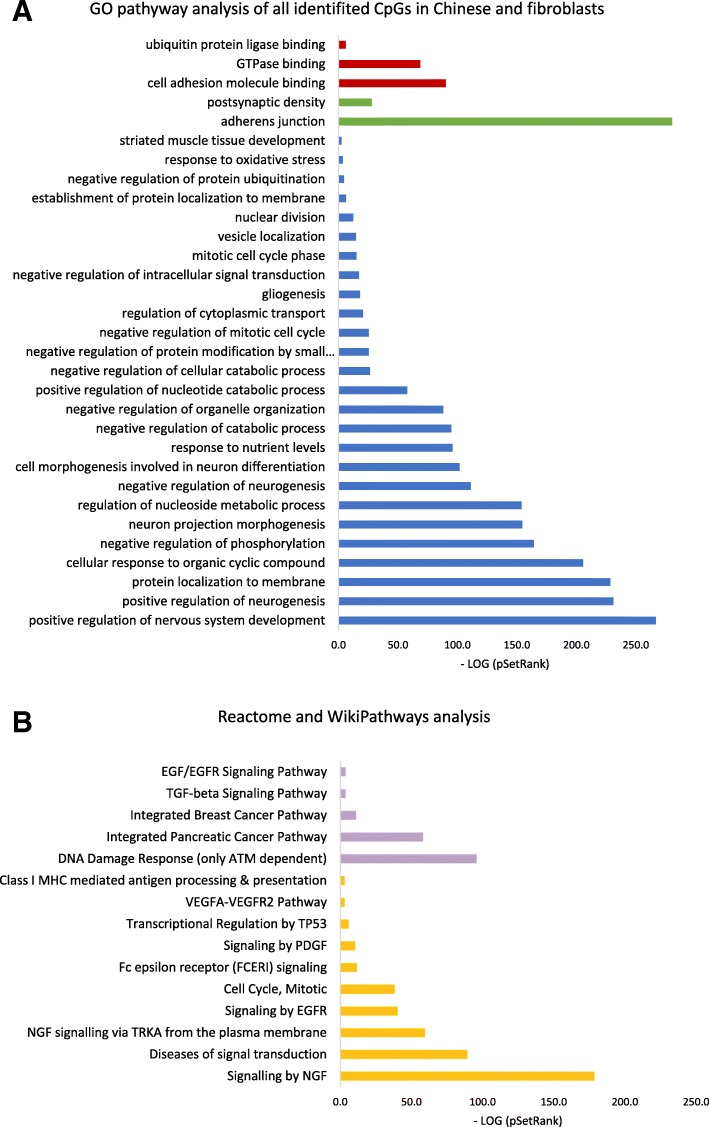
Significant pathway analysis based on CpGs (2706) associated with famine in both the Chinese famine and fibroblast study. **a** Significant pathways from GO analysis. Pathways in red represent molecular functions, in green represent cellular components, and in blue represent biological processes. *X*-axis displays the minus log *p* value of the association with the SetRank value of the gene set. **b** Significant pathway analysis from Reactome and WikiPathways. Reactome pathway is in purple, and WikiPathways is in orange. *X*-axis displays the minus log *p* value of association with the SetRank value of the gene set

Figure 1 shows the significant pathways that are associated with all the 2706 overlapping CpGs from the Chinese famine sample and fibroblast experiments. The pathway analysis is based on GO, KEGG, WikiPathways, and Reactome pathway databases. GO pathway analysis highlighted three significant molecular function pathways, among which cell adhesion molecule binding is mostly prevalent. Adherens junction is most relevant regarding the cellular components. In addition, we found that the famine condition influenced a wide range of biological processes, among which neuronal systems are most strongly implied. For example, pathways in nervous system development, both positive and negative neurogenesis, and neuron projection morphogenesis are highly involved. The pathway analysis from significant Reactome and WikiPathways analysis showed that DNA damage response and signaling by nerve growth factor (NGF) are mostly involved by nutritional deprivation.

## Discussion

This is the first study that combines genome-wide DNA methylation analysis of famine exposure with an in vitro study of nutritional deprivation to explore the effect of famine on DNA methylation. The results highlight several gene promoters that are differentially methylated due to nutritional deprivation. Further pathway analysis showed that the nervous system development and signaling by nerve growth factor (NGF) are sensitive to nutritional deprivation.

Analysis of the overlapping DMRs from Chinese famine samples and in vitro fibroblast samples identified three DMRs in four gene promoters (*ENO2*, *ZNF226*, *CCDC51,* and *TMA7*) that are consistently hypomethylated in relation to nutrition deprivation in both Chinese famine and fibroblast in vitro samples (Table 2). The fact that famine is consistently linked to hypomethylation and no occurrences of hypermethylation were identified suggests reduced methylation efficacy, for instance, due to the limited production of the methyl donor *S*-adenosyl methionine (SAMe) which is dependent on nutrients such as folate, vitamin B1, B6, and B12. Genes identified in the current study have a wide range of functions, but the involvement of the gene *ENO2* is one of the most interesting findings. *ENO2* is abundantly expressed neurons and peripheral neuroendocrine tissue [30] and often used as neuron-specific reference genes [31–33]. Functional studies showed that *ENO2* promotes cell proliferation, glycolysis, and glucocorticoid resistance [34], and silencing of this gene was found to inhibit the growth of glioblastoma cells [35]. Consistently, *ENO2* serves as a biochemical marker for tumors derived from neuronal and peripheral neuroendocrine tissues [34]. Furthermore, *ENO2* is found to be expressed higher in the brain of schizophrenia (SCZ) patient as compared to controls and may affect glucose metabolism in SCZ patients [36]. Moreover, a recent study found *ENO2* hypermethylation in autism alongside with decreased transcription and translation of this gene [37]. A look-up in BECon [38], an online database to compare the methylation pattern between brain and blood, suggests that part of the DMR in *ENO2* (cg08003732) has a similar DNA methylation pattern in blood and brain tissue.

Interpretation of the involvement of zinc finger protein gene *ZNF226* is less straightforward as not much is currently known about this specific gene. Zinc finger proteins have a broad range of molecular functions, and they are widely targeted for aberrant DNA hypermethylation during toxicant-induced malignant transformation [39] and as a driver of detrimental environment factor-associated carcinogenesis, leading to suggestions of their suitability for cancer prevention [40]. The third DMR identified, *CCDC51*, is a protein-coding gene, which is present in endosomes [41]. This gene is involved in several signaling pathways, such as B cell receptor activation [42], micronucleus formation regulation [43], cellular senescence [44], liver-specific microRNA binding [45], and tumor suppressor activity [46], as well as kidney disease [47]. Mouse *Ccdc51* gene is the target gene of miR-672-5p, which is highly expressed after steroid-induced osteonecrosis [48]. Considering that nutritional deprivation could potentially disturb steroid levels, the current finding of *CCDC51* hypomethylation raises the possibility of a relation between famine and steroid imbalance. The final DMR gene *TMA7* codes for the TMA7 protein, and deletion of this gene is consistent with loss of proteins involved in ribosome biogenesis [49]. Though the current finding is based on blood and fibroblasts, the database from Hannon et al. shows that methylation in blood of the four identified loci from the current study is correlated with the prefrontal cortex, five are correlated with the entorhinal cortex; two are correlated with the superior temporal gyrus, and three are correlated with the cerebellum. This suggests that blood methylation levels of these DMRs in part may serve as a proxy for methylation in these brain areas.

In the previous genome-wide methylation study of the Dutch hunger winter, 181 genes were identified through reduced representation bisulfite sequencing (RRBS) and a further 6 genes were verified in mass spectrometry-based EpiTYPER assay [8]. Later, in a Bangladesh famine cohort, seven epialleles were identified [4]. Although the DMRs from these previous studies do not overlap with our DMRs, the DMRs are near genes from the same pathway. For example, *ZNF251* and *CCDC57* were identified in the Dutch hunger cohort, whereas in our study, *ZNF226* and *CCDC51* are found differentially methylated. The different genetic background of the three famine cohort studies could be one of the explanations of these differences since the vulnerability to environmental factors could be inherent genetically [50]. Another explanation for the diverging results could be that although all three populations suffered from famine, the remaining food consumption pattern probably was quite different between countries. Differences in dietary nutrient intake could eventually lead to different patterns of malnutrition and to different outcomes.

The pathways most commonly related to malnutrition exposure are in the nervous system and neurogenesis, specifically, positive regulation of nervous system development in the GO pathway analysis (blue in Fig. 1) and nerve growth factor (NGF) signaling in the WikiPathways analysis (orange in Fig. 1). This points to the high relevance of epigenetic adaptations to famine for the brain [51] (even though current study did not analyze brain). Impact of famine on the brain has been shown in rodent studies that showed large epigenetic changes in the hippocampus in offsprings of nutritional deprived rats [52].

Performing DNA methylation analysis on fibroblasts in addition to whole blood increases the diversity of the tissue types and strongly reduces the risk that residual confounding factors are driving the results. Fibroblasts provide a different tissue type, and using longitudinal analysis within the same participants poses the opportunity to directly relate DNA methylation changes to famine. The replicating DMRs from fibroblasts and blood therefore provide compelling evidence that these are relevant genes that are involved in the response to malnutrition.

Some limitations should be considered when interpreting the current study. Replication of our findings in the Dutch famine [8] study was not possible due to the fact that these loci were eliminated in their analysis based on a low variance in whole-genome bisulphite sequencing data. Also, lookup of the presented DMRs in the studies of James et al. [53] and Finer et al. [4] did not identify an overlap. However, considering these studies essentially used candidate gene approaches in very different populations, this does not refute our findings. The merit of the current approach is the triangulation identifying epidemiological associations combined with an experimental biological response [54]. Inherent to the case-control setup of this study, other residual confounding factors such as for instance diet, cannot be ruled out. The identified DMRs from the current study are based on two different tissues and different experimental setups. Considering these differences, we expected a small number of overlapping DMRs from these two experiments. We expect that only truly strong biological effects will be detected in both experiments consistent with the concept of triangulation of research findings [54]. Nevertheless, the small overlaps between DMRs from both experiments underscore the limited similarity between studies and therefore have limited value as a replication. The sample sizes are relatively small, and therefore, power and significance level are limited. Also, although two tissue types were used, both the blood and fibroblast methylation may still not represent the situation in the developing brain. Finally, the genetic background from this study limits our conclusion on malnutrition response to Chinese and Dutch ancestry and may not represent other ethnic groups.

## Conclusions

Using an unbiased genome-wide approach, the current study examined the association between DNA methylation and severe nutritional deprivation in two unique samples separately (Chinese famine and in vitro fibroblasts) and leads to the identification of DMRs that were consistently hypomethylated in both samples. The three DMRs in the four gene promoters *ENO2*, *ZNF226*, *CCDC51*, and *TMA7* and the involvement of the nervous system development and signaling by nerve growth factor (NGF) that are suggested by pathway analyses can provide new leads to understand the pathways from nutrition deprivation to disease.

## Additional files

Additional file 1: A. QQ plot of the *p* value distribution for the regression of DNA methylation and Chinese famine samples. B. QQ plot of the *p* value distribution for the paired *t* test of DNA methylation and fibroblast in vitro samples. (PDF 58 kb)

Additional file 2: Nominally associated loci of Chinese and fibroblast samples. The file contains the loci ID and *p* value of each locus associated with nutritional deprivation in Chinese and fibroblast samples. (XLSX 4646 kb)

Additional file 3: Permutation analysis of DMRs. The file contains the results of the permutation analysis. (DOCX 14 kb)

## Abbreviations

CHR: Chromosome
CpG: Cytosine-phosphate-guanine
DMRs: Differentially methylated regions
DNA: Deoxyribonucleic acid
GO: Gene Ontology
GSEA: Gene Set Enrichment Analysis
KEGG: Kyoto Encyclopedia of Genes and Genomes
NGF: Nerve growth factor
RRBS: Reduced representation bisulfite sequencing

## References

[1] Schübeler D (2015). Function and information content of DNA methylation. Nature..

[2] Cho CE, Pannia E, Huot PSP, Sánchez-Hernández D, Kubant R, Dodington DW (2015). Methyl vitamins contribute to obesogenic effects of a high multivitamin gestational diet and epigenetic alterations in hypothalamic feeding pathways in Wistar rat offspring. Mol Nutr Food Res.

[3] Heijmans BT, Tobi EW, Stein AD, Putter H, Blauw GJ, Susser ES (2008). Persistent epigenetic differences associated with prenatal exposure to famine in humans. Proc Natl Acad Sci U S A.

[4] Finer S, Iqbal MS, Lowe R, Ogunkolade BW, Pervin S, Mathews C (2016). Is famine exposure during developmental life in rural Bangladesh associated with a metabolic and epigenetic signature in young adulthood? A historical cohort study. BMJ Open.

[5] Huang C, Li Z, Wang M, Martorell R (2010). Early Life Exposure to the 1959-1961 Chinese famine has long-term health consequences. J Nutr.

[6] Tobi EW, Slieker RC, Luijk R, Dekkers KF, Stein AD, Xu KM (2018). DNA methylation as a mediator of the association between prenatal adversity and risk factors for metabolic disease in adulthood. Sci Adv.

[7] Lumey LH, Stein AD, Kahn HS, Van der Pal-de Bruin KM, Blauw GJ, Zybert PA (2007). Cohort profile: the Dutch Hunger Winter families study. Int J Epidemiol.

[8] Tobi EW, Goeman JJ, Monajemi R, Gu H, Putter H, Zhang Y (2014). DNA methylation signatures link prenatal famine exposure to growth and metabolism. Nat Commun.

[9] Boks MPM, Houtepen CL, Xu Z, He Y, Ursini G, Maihofer A (2018). Genetic vulnerability to DUSP22 promotor hypermethylation is involved in the relation between in utero famine exposure and schizophrenia. NPJ Schizophr..

[10] Vaiserman AM. Early-life nutritional programming of type 2 diabetes: Experimental and quasi-experimental evidence. Nutrients. 2017. 10.3390/nu9030236 PMC5372899 28273874

[11] Ashton B, Hill K, Piazza A, Zeitz R. Famine in China, 1958-61. Popul Dev Rev. 1984;10.

[12] Fan W, Qian Y (2015). Long-term health and socioeconomic consequences of early-life exposure to the 1959-1961 Chinese famine. Soc Sci Res..

[13] Li C, Lumey LH (2017). Exposure to the Chinese famine of 1959-61 in early life and long-term health conditions: a systematic review and meta-analysis. Int J Epidemiol..

[14] Sun Y, Zhang L, Duan W, Meng X, Jia C (2018). Association between famine exposure in early life and type 2 diabetes mellitus and hyperglycemia in adulthood: results from the China Health And Retirement Longitudinal Study (CHARLS). J Diabetes.

[15] Xu MQ, Sun WS, Liu BX, Feng GY, Yu L, Yang L (2009). Prenatal malnutrition and adult schizophrenia: further evidence from the 1959-1961 Chinese famine. Schizophr Bull..

[16] St Clair D, Xu M, Wang P, Yu Y, Fang Y, Zhang F (2005). Rates of adult schizophrenia following prenatal exposure to the Chinese famine of 1959-1961. J Am Med Assoc..

[17] Boks MP, Houtepen LC, Xu Z, He Y, Ursini G, Maihofer AX (2018). Genetic vulnerability to DUSP22 promoter hypermethylation is involved in the relation between in utero famine exposure and schizophrenia. NPJ Schizophr.

[18] Aryee MJ, Jaffe AE, Corrada-Bravo H, Ladd-Acosta C, Feinberg AP, Hansen KD (2014). Minfi: A flexible and comprehensive Bioconductor package for the analysis of Infinium DNA methylation microarrays. Bioinformatics. Department of Pathology, Massachusetts General Hospital and Harvard Medical School, Boston, MA 02114, USA, Department of Biostatistics, Johns Hopkins School of Public Health, 615 N Wolfe Street, Baltimore, MD 21205, USA. Lieber Institute of Brain Developm.

[19] Barfield RT, Almli LM, Kilaru V, Smith AK, Mercer KB, Duncan R (2014). Accounting for population stratification in DNA methylation studies. Genet Epidemiol..

[20] Chen YA, Lemire M, Choufani S, Butcher DT, Grafodatskaya D, Zanke BW (2013). Discovery of cross-reactive probes and polymorphic CpGs in the Illumina Infinium HumanMethylation450 microarray. Genetics and Genome Biology Program, Hospital for Sick Children, Toronto, ON, Canada. Epigenetics.

[21] Houseman EA, Accomando WP, Koestler DC, Christensen BC, Marsit CJ, Nelson HH (2012). DNA methylation arrays as surrogate measures of cell mixture distribution. BMC Bioinformatics..

[22] Min J, Hemani G, Smith GD, Relton CL, Suderman M. Meffil: efficient normalisation and analysis of very large DNA methylation samples. bioRxiv. Cold Spring Harbor Laboratory; 2017;125963.

[23] Fortin J-P, Labbe A, Lemire M, Zanke BW, Hudson TJ, Fertig EJ (2014). Functional normalization of 450 k methylation array data improves replication in large cancer studies. Genome Biol..

[24] Du P, Zhang X, Huang C-C, Jafari N, Kibbe WA, Hou L, et al. Comparison of beta-value and M-value methods for quantifying methylation levels by microarray analysis. Northwestern University Biomedical Informatics Center (NUBIC), NUCATS, Feinberg School of Medicine, Northwestern University, Chicago, IL 60611, USA. dupan@northwestern.edu; BMC Bioinformatics. 2010;11:587. 10.1186/1471-2105-11-587 PMC3012676 21118553

[25] Simillion C, Liechti R, Lischer HEL, Ioannidis V, Bruggmann R (2017). Avoiding the pitfalls of gene set enrichment analysis with SetRank. BMC Bioinformatics..

[26] R Core Team. R Core Team (2014). R: a language and environment for statistical computing. R Found. Stat. Comput. Vienna: R Foundation for Statistical Computing; 2014. http://www.R-project.org/.

[27] Hannon E, Dempster E, Viana J, Burrage J, Smith AR, Macdonald R (2016). An integrated genetic-epigenetic analysis of schizophrenia: evidence for co-localization of genetic associations and differential DNA methylation. Genome Biol BioMed Central.

[28] Peters TJ, Buckley MJ, Statham AL, Pidsley R, Samaras K, V Lord R (2015). De novo identification of differentially methylated regions in the human genome. Epigenetics Chromatin..

[29] Hannon E, Lunnon K, Schalkwyk LC, Mill J. Interindividual methylomic variation across blood, cortex, and cerebellum: implications for epigenetic studies of neurological and neuropsychiatric phenotypes. Epigenetics. a University of Exeter Medical School, RILD Building (Level 4) , Barrack Road, University of Exeter , Devon , UK a University of Exeter Medical School, RILD Building (Level 4) , Barrack Road, University of Exeter , Devon , UK b School of Biological Scienc; 2015. 10.1080/15592294.2015.1100786 PMC4844197 26457534

[30] Craig SP, Day INM, Thompson RJ, Craig IW (1990). Localisation of neurone-specific enolase (ENO2) to 12pl3. Cytogenet Genome Res.

[31] Gatta E, Auta J, Gavin DP, Bhaumik DK, Grayson DR, Pandey SC (2017). Emerging role of one-carbon metabolism and DNA methylation enrichment on delta-containing GABAA receptor expression in the cerebellum of subjects with alcohol use disorders (AUD). Int J Neuropsychopharmacol.

[32] Guidotti A, Auta J, Davis JM, Gerevini VD, Dwivedi Y, Grayson DR, et al. Decrease in reelin and glutamic acid decarboxylase67 (GAD67) expression in schizophrenia and bipolar disorder. Arch Gen Psychiatry. 2000. 10.1001/archpsyc.57.11.1061 11074872

[33] Teocchi MA, Ferreira AE, da Luz de Oliveira EP, Tedeschi H, D’Souza-Li L (2013). Hippocampal gene expression dysregulation of Klotho, nuclear factor kappa B and tumor necrosis factor in temporal lobe epilepsy patients. J Neuroinflammation.

[34] Liu C-C, Wang H, Wang W, Wang L, Liu W-J, Wang J-H (2018). ENO2 promotes cell proliferation, glycolysis, and glucocorticoid-resistance in acute lymphoblastic leukemia. Cell Physiol Biochem.

[35] Muller FL, Colla S, Aquilanti E, Manzo VE, Genovese G, Lee J (2012). Passenger deletions generate therapeutic vulnerabilities in cancer. Nature..

[36] Martins-de-Souza D, Gattaz WF, Schmitt A, Novello JC, Marangoni S, Turck CW (2009). Proteome analysis of schizophrenia patients Wernicke’s area reveals an energy metabolism dysregulation. BMC Psychiatry..

[37] Wang Y, Fang Y, Zhang F, Xu M, Zhang J, Yan J (2014). Hypermethylation of the enolase gene (ENO2) in autism. Eur J Pediatr.

[38] Edgar RD, Jones MJ, Meaney MJ, Turecki G, Kobor MS (2017). BECon: a tool for interpreting DNA methylation findings from blood in the context of brain. Transl Psychiatry.

[39] Severson PL, Tokar EJ, Vrba L, Waalkes MP, Futscher BW (2013). Coordinate H3K9 and DNA methylation silencing of ZNFs in toxicant-induced malignant transformation. Epigenetics..

[40] Rao CV, Asch AS, Yamada HY (2017). Frequently mutated genes/pathways and genomic instability as prevention targets in liver cancer. Carcinogenesis. England.

[41] Gosney JA, Wilkey DW, Merchant ML, Ceresa BP (2018). Proteomics reveals novel protein associations with early endosomes in an epidermal growth factor-dependent manner. J Biol Chem.

[42] Schrader A, Meyer K, Walther N, Stolz A, Feist M, Hand E (2016). Identification of a new gene regulatory circuit involving B cell receptor activated signaling using a combined analysis of experimental, clinical and global gene expression data. Oncotarget..

[43] McIntyre RE, Nicod J, Robles-Espinoza CD, Maciejowski J, Cai N, Hill J (2016). A genome-wide association study for regulators of micronucleus formation in mice. G3 (Bethesda).

[44] Lopez MF, Niu P, Wang L, Vogelsang M, Gaur M, Krastins B (2017). Opposing activities of oncogenic MIR17HG and tumor suppressive MIR100HG clusters and their gene targets regulate replicative senescence in human adult stem cells. NPJ Aging Mech Dis.

[45] Fan B, Sutandy FXR, Syu G-D, Middleton S, Yi G, Lu K-Y (2015). Heterogeneous ribonucleoprotein K (hnRNP K) binds miR-122, a mature liver-specific microRNA required for hepatitis C virus replication. Mol. Cell. Proteomics..

[46] D’Agostino S, Lanzillotta D, Varano M, Botta C, Baldrini A, Bilotta A (2018). The receptor protein tyrosine phosphatase PTPRJ negatively modulates the CD98hc oncoprotein in lung cancer cells. Oncotarget..

[47] Schmidts M, Frank V, Eisenberger T, al Turki S, Bizet AA, Antony D (2013). Combined NGS approaches identify mutations in the intraflagellar transport gene IFT140 in skeletal ciliopathies with early progressive kidney disease. Hum Mutat.

[48] Li P, Sun N, Zeng J, Zeng Y, Fan Y, Feng W (2016). Differential expression of miR-672-5p and miR-146a-5p in osteoblasts in rats after steroid intervention. Gene Elsevier BV.

[49] Fleischer TC, Weaver CM, McAfee KJ, Jennings JL, Link AJ (2006). Systematic identification and functional screens of uncharacterized proteins associated with eukaryotic ribosomal complexes. Genes Dev..

[50] Schoenrock SA, Oreper D, Farrington J, Mcmullan RC, Ervin R, Miller DR, et al. Perinatal nutrition interacts with genetic background to alter behavior in a parent-of-origin-dependent manner in adult Collaborative Cross mice. Genes Brain Behav. 2017. 10.1111/gbb.12438 PMC6705147 29125223

[51] Delgado-Morales R, Agís-Balboa RC, Esteller M, Berdasco M (2017). Epigenetic mechanisms during ageing and neurogenesis as novel therapeutic avenues in human brain disorders. Clin Epigenetics..

[52] Xu J, He G, Zhu J, Zhou X, Clair DS, Wang T (2014). Prenatal nutritional deficiency reprogrammed postnatal gene expression in mammal brains: Implications for schizophrenia. Int J Neuropsychopharmacol..

[53] James P, Sajjadi S, Tomar AS, Saffari A, Fall CHD, Prentice AM (2018). Candidate genes linking maternal nutrient exposure to offspring health via DNA methylation: a review of existing evidence in humans with specific focus on one-carbon metabolism. Int J Epidemiol..

[54] Munafò MR, Davey Smith G (2018). Robust research needs many lines of evidence. Nature..

