# Supplementary figures and images for: DNA methylation changes related to nutritional deprivation: a genome-wide analysis of population and in vitro data
Source: Clin Epigenetics. 2019 May 16;11:80. doi: 10.1186/s13148-019-0680-7 (PMC6524251; doi:10.1186/s13148-019-0680-7)

A.

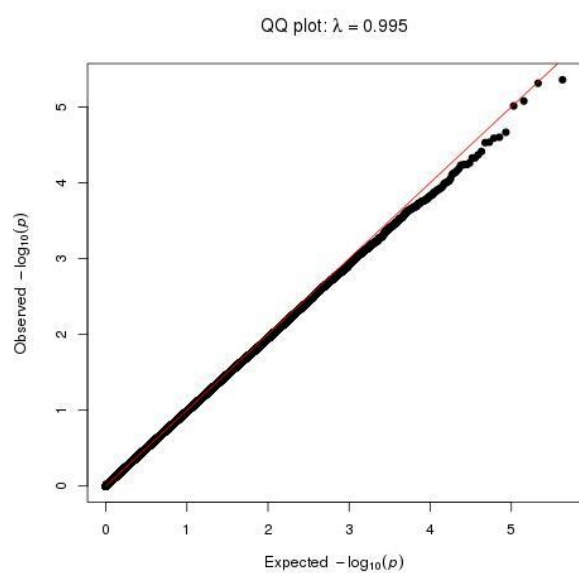

B.

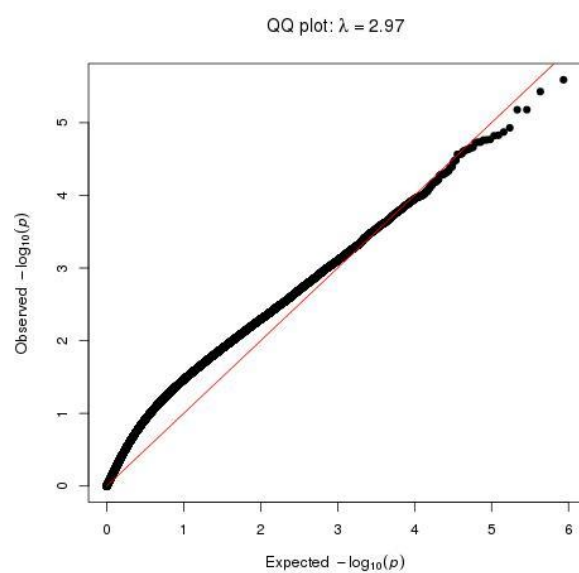

Supplement: Supplementary file 1 — A. QQ plot of the p value distribution for the regression of DNA methylation and Chinese famine samples. B. QQ plot of the p value distribution for the paired t test of DNA methylation and fibroblast in vitro samples. (PDF 58 kb) [file 13148_2019_680_MOESM1_ESM.pdf]
