# Supplementary material for: DNA methylation changes related to nutritional deprivation: a genome-wide analysis of population and in vitro data
Source: Clin Epigenetics. 2019 May 16;11:80. doi: 10.1186/s13148-019-0680-7 (PMC6524251; doi:10.1186/s13148-019-0680-7)
Supplement: Supplementary file 3 — Permutation analysis of DMRs. The file contains the results of the permutation analysis. (DOCX 14 kb) [file 13148_2019_680_MOESM3_ESM.docx]

Table S3: Permutation analysis DMR’s

| DMR | Symbol | Chrom | nr cpgs | P-value  Fibroblast | P-value  Chineese | Combined  (Stouffer) | Probability of overlap |
| --- | --- | --- | --- | --- | --- | --- | --- |
| 2 | ENO2 | 12 | 4 | 0.0223 | 0.0999 | 1.58e-2 | <0.0001 |
| 4 | ZNF226 | 19 | 5 | 0.0429 | 0.0357 | 1.14e-2 | <0.0001 |
| 8 | CCDC51 | 3 | 13 | 0.0143 | 0.0027 | 4.34e-4 | <0.0001 |

DMR: DMRnummer

Chrom: chromosome

nr cpgs: number of cpgs in the DMR

P-value Fibroblast: Emperical p-value of the DMR in the fibroblast experiment

P-value Chineese: Emperical p-value of the DMR in the Chinese famine data

Combined: combined p-value calculated using stouffer’s method

Probability of overlap: probability of finding one overlapping DMR between experiments.
